# Supplementary material for: The SARS-CoV-2 Omicron BA.1 spike G446S mutation potentiates antiviral T-cell recognition
Source: Nat Commun. 2022 Sep 21;13:5440. doi: 10.1038/s41467-022-33068-4 (PMC9492656; doi:10.1038/s41467-022-33068-4)
Supplement: Supplementary file 1 — Supplementary Information [file 41467_2022_33068_MOESM1_ESM.pdf]

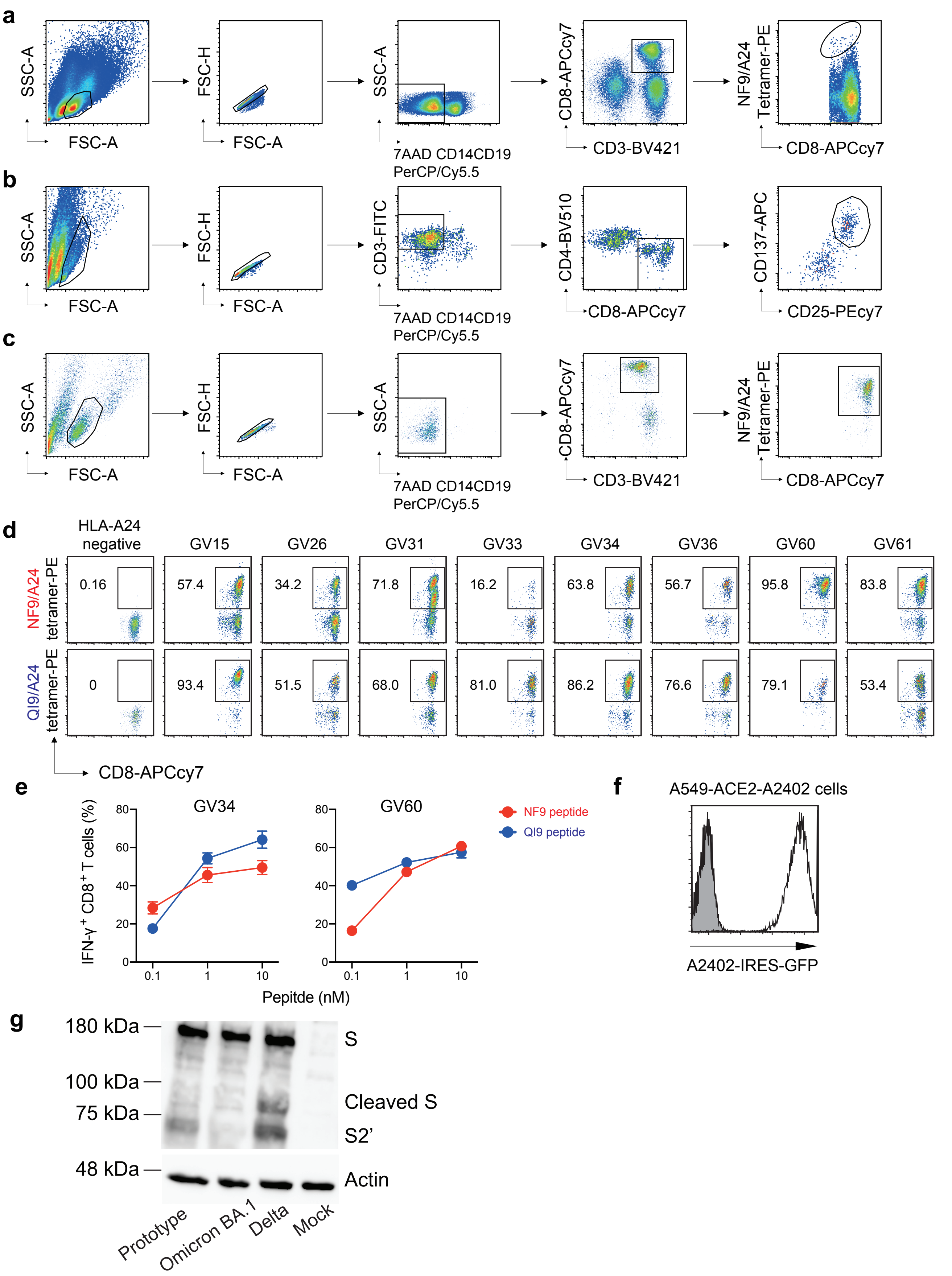

Supplementary Figure 1. Gating strategy and T cell and cell lines used in this study.

(a-c) Flow cytometry gating strategy of tetramer staining in PBMCs from donor GV16 (a), CD25$^+$CD137$^+$ activated T cell lines from donor GV60 (b) and tetramer staining of T cell lines from GV60 (c). (d) NF9 and QI9 stimulated-T cell lines used in this study. (e) IFN-γ production in peptide titration of the NF9 and QI9 peptide in T cell lines from GV34 and GV60 donors. The assay was performed in triplicate, and the means are shown with the SD. (f) GFP expression of A549-ACE2-A2402-IRES-GFP cells. A549-ACE2 parental (shaded histogram) and FACS-sorted A549-ACE2-A2402-IRES-GFP cells (open histogram) are shown. (g) Western blot. Representative blots of cells expressing prototype, Omicron BA.1, and Delta spike (S) protein of three independent experiments. Source data are provided as a Source Data file.

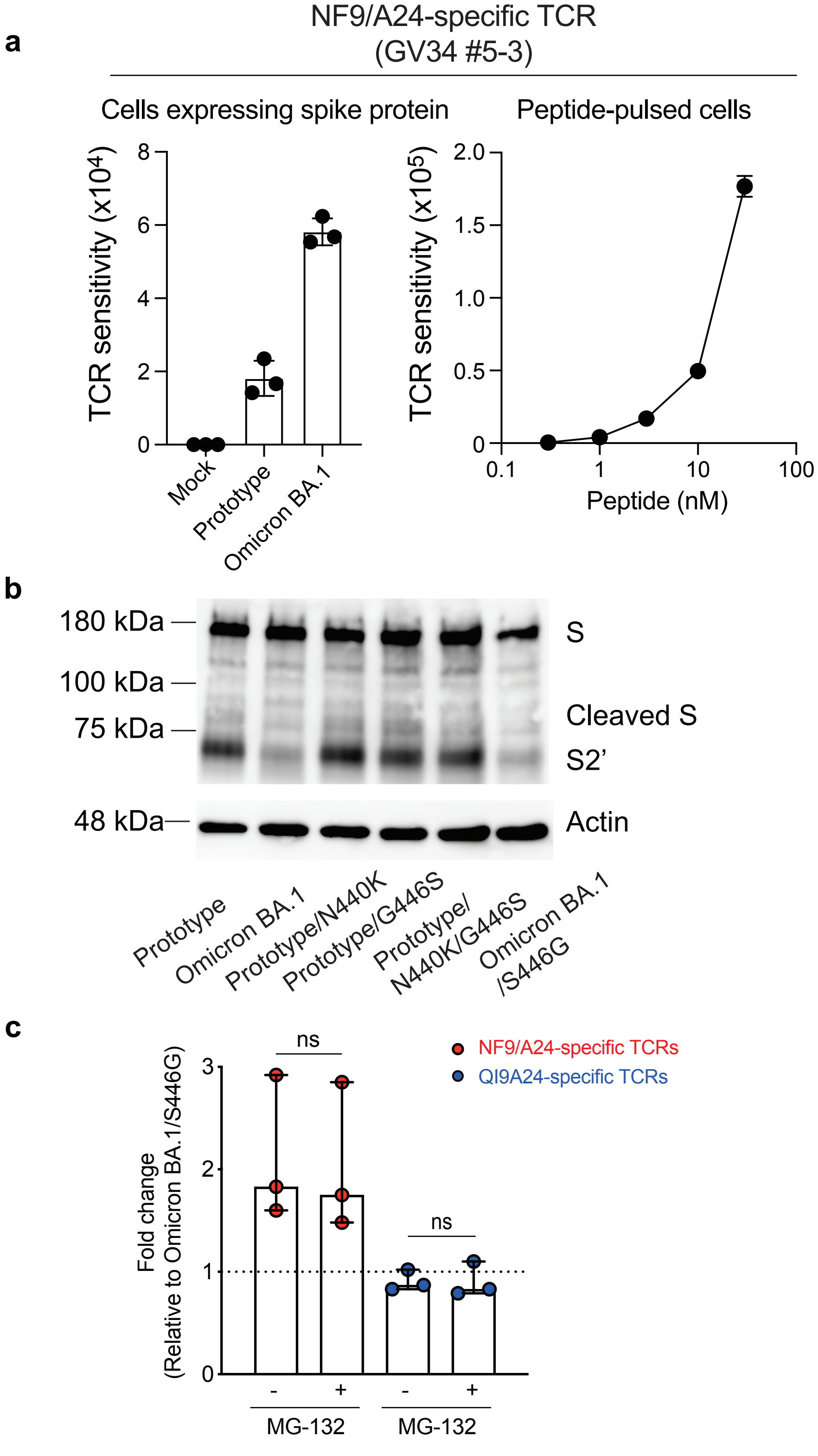

Supplementary Figure 2. Cell lines used in TCR-sensitivity assay.

(a) TCR sensitivity of GV34 #5-3 toward cells expressing spike protein and peptide-pulsed target cells.

(b) Representative western blots of cells expressing various spike (S) proteins of three independent experiments. Source data are provided as a Source Data file. (c) The level of peptide/HLA complexes was evaluated following treatment with a proteasome inhibitor, MG-132 (10 μM). The effect of the inhibitor was evaluated as reporter activity and shown as a fold change in the level of peptide/HLA on target cells expressing Omicron BA.1 spike protein with the S446G reversion (Omicron BA.1/S446G). Statistical analysis versus DMSO alone was determined by an unpaired two-tailed Student' s t-test. Data are expressed as a median. ns, no statistical significance. a, c The assay was performed in triplicate, and the means are shown with the SD. Data are representative of three independent experiments.

Supplementary Table 1. Human PBMCs used in this study, related to Fig. 1

Donor ID	Sex	Age	HLA-A24?	vaccinated?	Days after two doses of vaccination
Vku19	Female	36	Positive	BNT162b2	21
GV1	Female	28	Positive	BNT162b2	9
GV4	Female	18	Negative	BNT162b2	20
GV5	Female	18	Positive	BNT162b2	20
GV6	Female	79	Positive	BNT162b2	21
GV9	Female	24	Positive	BNT162b2	21
GV10	Male	23	Positive	BNT162b2	22
GV11	Male	23	Positive	BNT162b2	22
GV12	Female	28	Negative	BNT162b2	23
GV13	Male	22	Positive	BNT162b2	21
GV14	Male	23	Positive	BNT162b2	22
GV15	Female	23	Positive	BNT162b2	21
GV16	Male	22	Positive	BNT162b2	22
GV17	Male	24	Negative	BNT162b2	21
GV19	Male	24	Positive	BNT162b2	22
GV20	Female	24	Positive	BNT162b2	22
GV21	Male	22	Positive	BNT162b2	21
GV22	Male	22	Positive	BNT162b2	22
GV23	Male	25	Positive	BNT162b2	22
GV24	Male	23	Positive	BNT162b2	22
GV25	Male	24	Negative	BNT162b2	22
GV26	Male	23	Positive	BNT162b2	22
GV27	Female	23	Negative	BNT162b2	21
GV28	Female	23	Positive	BNT162b2	21
GV29	Male	23	Positive	BNT162b2	21
GV31	Male	23	Positive	BNT162b2	22
GV32	Male	56	Positive	BNT162b2	27
GV33	Male	39	Positive	BNT162b2	24
GV34	Female	38	Positive	BNT162b2	24
GV35	Male	52	Positive	BNT162b2	24
GV36	Male	39	Positive	BNT162b2	24
GV51	Male	34	Positive	BNT162b2	30
GV59	Male	37	Positive	BNT162b2	126
GV60	Male	51	Positive	mRNA-1273	116
GV61	Female	36	Positive	mRNA-1273	107

Supplementary Table 2. TCR sequences isolated from NF9/A24 and QI9/A24 specific T cells by single-cell analysis, related to Fig. 3

Specificity	ID	TRAV	TRAJ	CDR3α	TRBV	TRBJ	TRBD	CDR3β
NF9/A24	GV34 #2-2	TRAV12-1*01	TRAJ33*01	CVVNALMDSNYQLIW	TRBV5-1*01	TRBJ2-7*01	TRBD1*01	CASSLGQGYEQYF
	GV34 #5-3	TRAV12-1*01	TRAJ33*01	CVVNLFDSNYQLIW	TRBV2*01	TRBJ2-7*01	TRBD1*01	CASSEGAGYEQYF
	VKU19 #12-3	TRAV12-3*01	TRAJ44*01	CAFTGTASKLTF	TRBV7-8*01	TRBJ2-1*01	TRBD2*02	CASSPELNEQFF
QI9/A24	GV33 #57	TRAV3*01	TRAJ8*01	CAGVLFNTGFQKLVF	TRBV20-1*02	TRBJ2-1*01	TRBD2*01	CSASDRGASGSFSNEQFF
	GV34 #43	TRAV21*01	TRAJ43*01	CAAPRYNNNDMRF	TRBV2*01	TRBJ2-2*01	TRBD1*01	CASSEGADAGELFF
	GV36 #10-2	TRAV19*01	TRAJ9*01	CALSEPPSGGFKTIF	TRBV20-1*01	TRBJ1-1*01	TRBD1*01	CSARGQGLNTEAFF

Supplementary Table 3. Primers for the construction of spike derivatives, related to Fig. 3

Product		Primer name	Sequence (5'-to-3')
Prototype spike	N440K	N440K Fwd	CAGCAACAAGCTGGACAGCAAGGTGG
		N440K Rev	CTGTCCAGCTTGTTGCTGTTCCAGGC
	N440K G446S	N440K G446S Fwd	GCAACAAGCTGGACAGCAAGGTGTCCGGCAACTACAAC
		N440K G446S Rev	GTTGCCGGACACCTTGCTGTCCAGCTTGTTGCTGTTC
	G446S	G446S Fwd	CAAGGTGTCCGGCAACTACAACTACCTC
		G446S Rev	GTAGTTGCCGGACACCTTGCTGTCCAG
Omicron BA.1 spike	S446G	S446G Fwd	GACAGCAAGGTGGGAGGCAACTACA
		S446G Rev	GCCTCCCACCTTGCTGTCCAGCTTG
